# Frequency-specific electromagnetic modulation of brain redox state precedes cortical activity in stroke recovery

**DOI:** 10.1016/j.isci.2026.114731

**Published:** 2026-01-19

**Authors:** Henry Hing Cheong Lee, Shira Reznik Balter, Nathaniel W. Hodgson, Batsheva Weisinger, Esther Shohami, Glen M. Doniger, Ana Parabucki, Yaron Segal, Takao K. Hensch

**Affiliations:** 1 FM Kirby Neurobiology Center, Department of Neurology, Boston Children’s Hospital, 300 Longwood Avenue, Boston, MA 02115, USA; 2 Rosamund Stone Zander Translational Neuroscience Center, Boston Children’s Hospital, 3 Blackfan Circle, Boston, MA 02115, USA; 3 BrainQ Technologies Ltd., Givat Ram Hi-Tech Village, Booth 3.7, Jerusalem, Israel; 4 Institute for Drug Research, Hebrew University of Jerusalem, Jerusalem, Israel; 5 International Research Center for Neurointelligence (IRCN), University of Tokyo Institutes for Advanced Study, Tokyo 113, Japan; 6 Center for Brain Science, Department of Molecular Cellular Biology, Harvard University, 52 Oxford Street, Cambridge, MA 02138, USA

**Keywords:** Biotechnology, applied sciences, Engineering

## Abstract

Mitigating oxidative stress is essential for recovery from stroke and other brain pathologies. Transient modulation of reactive oxygen species production triggers both cellular defense/repair mechanisms and neuroplasticity. Extremely low-frequency, low-intensity electromagnetic field (ELF-EMF) exposure may achieve both non-invasively. Here, we explored longitudinal consequences of frequency-specific ELF-EMF treatment on oxidative stress and cortical electrical activity in mice, and the implications for stroke recovery in humans. In mice, chronic (4w), daily 40Hz ELF-EMF (3h) exposure was neuroprotective across brain regions, as reflected in glutathione oxidation state. Notably, this followed an initial redox burden at treatment onset that was also detectable peripherally (urinary isoprostane, IsoP). Cortical rhythms emerged only gradually after chronic treatment. Enhanced EEG beta (13-30 Hz) power correlated with initial IsoP levels in mice and the degree of stroke recovery in humans. Taken together, ELF-EMF induces a sequential redox state dynamic that drives cortical activity toward functional recovery.

## Highlights

• Efficacy of ELF-EMF stimulation is frequency-specific
• Chronic ELF-EMF is neuroprotective after an initial redox imbalance
• Cortical rhythmicity strengthens gradually only after chronic ELF-EMF
• Enhanced β/γ-power correlates with initial redox burden and human stroke recovery

## Graphical abstract

**Figure:**
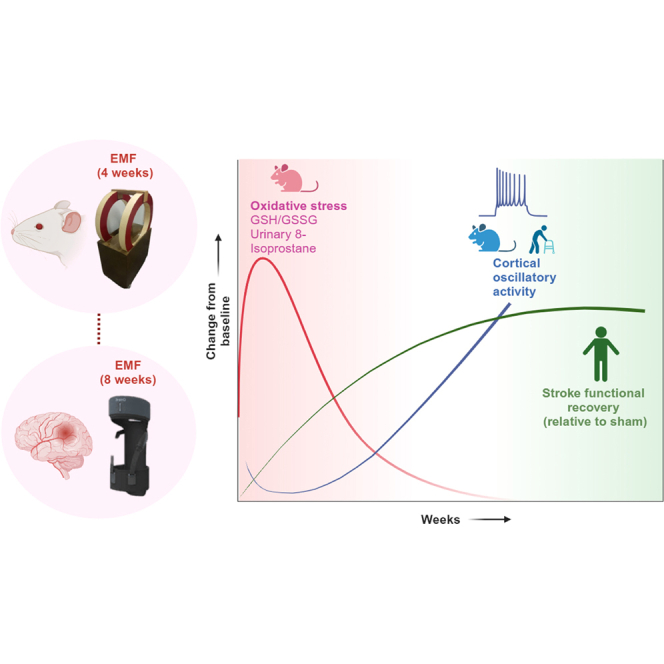

## Introduction

Oxidative stress is a significant player in the pathophysiology of various neurodegenerative disorders, including ischemic stroke. The resulting cellular damage from secondary injury processes often outpaces the limited intrinsic recovery process in these conditions. One approach to mitigate the symptoms associated with oxidative stress involves pharmacotherapy aimed to enhance the antioxidative capacity of the system and increase the removal of reactive oxygen species (ROS) from the brain. However, there are significant limitations, such as the brain accessibility of these therapeutic agents, pharmacokinetics, and potential systemic side effects, which can hinder the effectiveness of this strategy.^1^

Non-invasive neuromodulatory techniques present a potential alternative to pharmacotherapy. These techniques may decrease oxidative stress and produce appreciable therapeutic effects, offering non-invasive and accessible treatment for patients. Extremely low-frequency, electromagnetic field (ELF-EMF) exposure is one such technique that has shown promise in reducing oxidative stress markers in patients post-stroke.^2^ Furthermore, ELF-EMF, with wavelengths between 1 and 300 Hz, has been associated with reductions in stroke severity and improvements in cognitive and behavioral outcomes.^3^

ELF-EMF has not only been found to stimulate the innate immune response^4^^,^^5^ and osteogenesis,^6^^,^^7^ but it also shows promise in promoting recovery from stroke^8^^,^^9^ and spinal cord injury.^10^ Despite the low intensity (∼μT) of ELF-EMF establishing its safety profile as an emerging neuroprotective technique with demonstrated capacity to support neuroplastic processes,^11^^,^^12^^,^^13^ the underlying biological mechanisms of its impact within the intact brain remain unclear. To optimize treatment parameters such as stimulus frequency, duration, and interval for therapeutic purposes, a deeper understanding of how ELF-EMF interacts with the brain’s biochemistry and electrophysiology is needed.

In this study, we report that ELF-EMF exposure in naive, freely behaving mice first leads to brain-wide redox regulation. Notably, the acute oxidative burden mediated by ELF-EMF is frequency-specific and reverses with the increased duration of exposure. These redox dynamics are followed by functional changes in electrical activity, detectable via power spectral analyses of the electroencephalogram (EEG). Furthermore, these results parallel those of patients with subacute ischemic stroke, exhibiting abnormal EEG spectral power at baseline, whose clinical improvement is correlated with an increase in beta oscillations over the course of the ELF-EMF treatment. This highlights a frequency- and time-dependent, brain-wide redox equilibration by ELF-EMF that distinguishes it from other common forms of region-specific neuromodulation (e.g., Transcranial Alternating Current Stimulation (tACS), or Transcranial Magnetic Stimulation (TMS)).

## Results

### Dynamic brain-wide redox regulation by electromagnetic field is frequency-specific

Adult mice were exposed to 40 Hz ELF-EMF for 3 h daily over a 4-week treatment period. After the last session, mice were sacrificed and their brains dissected for biochemistry. Using high-performance liquid chromatography (HPLC), we analyzed the content of glutathione in its reduced (GSH) versus oxidized (GSSG) form, as a measure of cellular oxidative stress.

**Figure 1:**
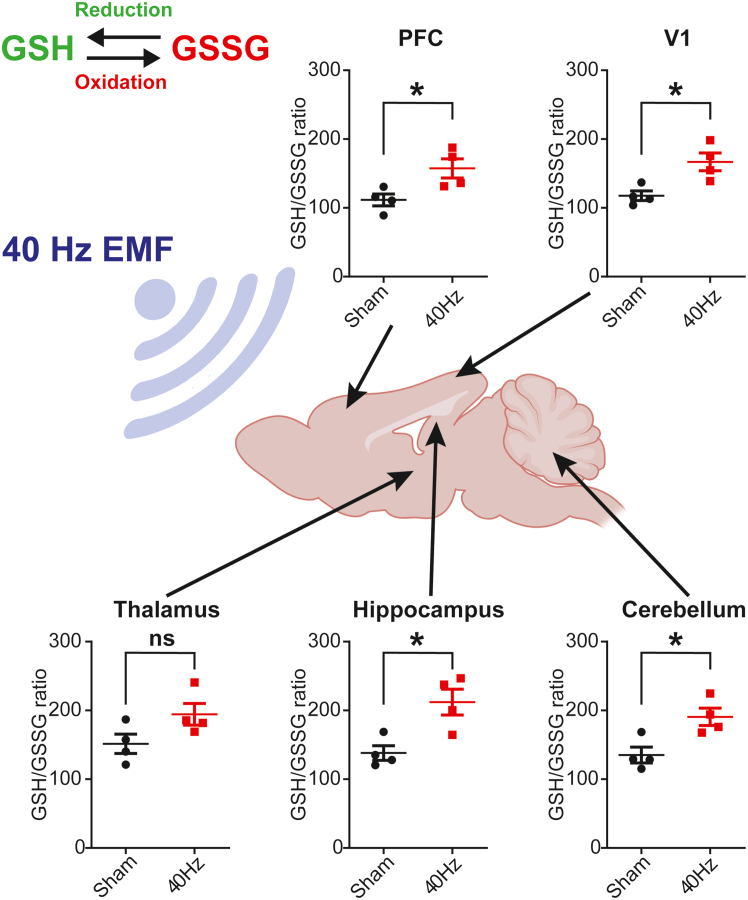
Chronic (4 weeks) ELF-EMF reduces brain-wide oxidative stress Cellular oxidative stress as determined by the ratio of glutathione in its reduced (GSH) versus oxidized form (GSSG) (i.e., GSH/GSSG Ratio). High-performance liquid chromatography (HPLC) of homogenized micro-dissected mouse brain tissue, including prefrontal cortex (PFC), primary visual cortex (V1), thalamus, hippocampus, and cerebellum. High GSH/GSSG ratio indicates low oxidative stress. Mice were exposed to 40 Hz ELF-EMF (10G) for 3 h daily over 4 weeks (except weekends) before tissue dissection. Data presented as Mean ± S.E.M. (∗*p* < 0.05, ns = not significant, *n* = 4, unpaired *t* test).

**Figure 2:**
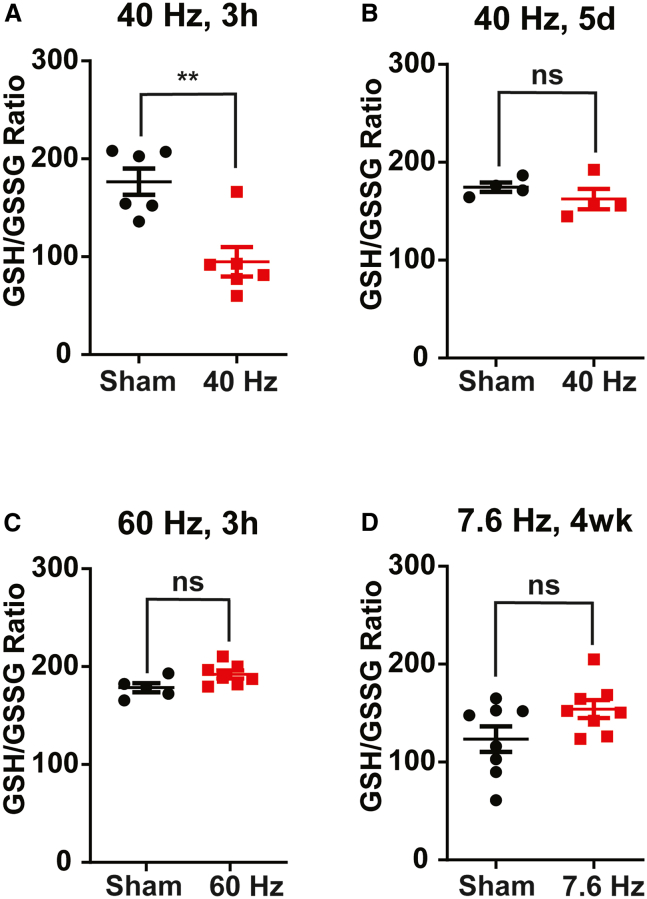
Transient redox dynamics and frequency specificity of ELF-EMF Brain cellular oxidative stress is measured and represented similarly to that in Figure 1. Mice were treated with ELF-EMF (10G) for a variety of frequencies and durations. (A) 40 Hz, 3 h, (B) 40 Hz, 5 days. (C) 60 Hz, 3 h, or (D) 7.6 Hz, 4 weeks (daily). Data presented as Mean ± S.E.M. (∗∗*p* < 0.01, ns = not significant, n = 4–8, unpaired *t* test).

Antioxidant capacity, such as that related to reduced and oxidized glutathione GSH/GSSG ratio, was increased in ELF-EMF-exposed mice compared to sham exposure (Figure 1), suggesting overall decreased oxidative stress throughout brain regions. To track the dynamics of ELF-EMF-induced cellular oxidative stress, we exposed separate cohorts of mice to various ELF-EMF durations and frequencies prior to brain removal as described above. In contrast to the effects of long-term exposure, we found that acute (3 h) ELF-EMF exposure increased oxidative stress (decreased GSH/GSSG ratio) in the cortex, while 5 days of ELF-EMF exposure did not alter GSH/GSSG substantially (Figures 2A–2B). Combined with the results from Figure 1, ELF-EMF thus produces bidirectional changes in cellular oxidative stress in a time-dependent manner. Moreover, the acute oxidative stress effects upon initial ELF-EMF exposure appear to be frequency-specific, as 60 Hz did not produce the same GSH/GSSG reduction as seen for 40 Hz (Figures 2D–2E). Furthermore, chronic (4-week) exposure to lower frequency (7.6 Hz) ELF-EMF did not significantly change the GSH/GSSG ratio (Figure 2F).

### Enhanced neural activity follows redox dysregulation by the electromagnetic field

**Figure 3:**
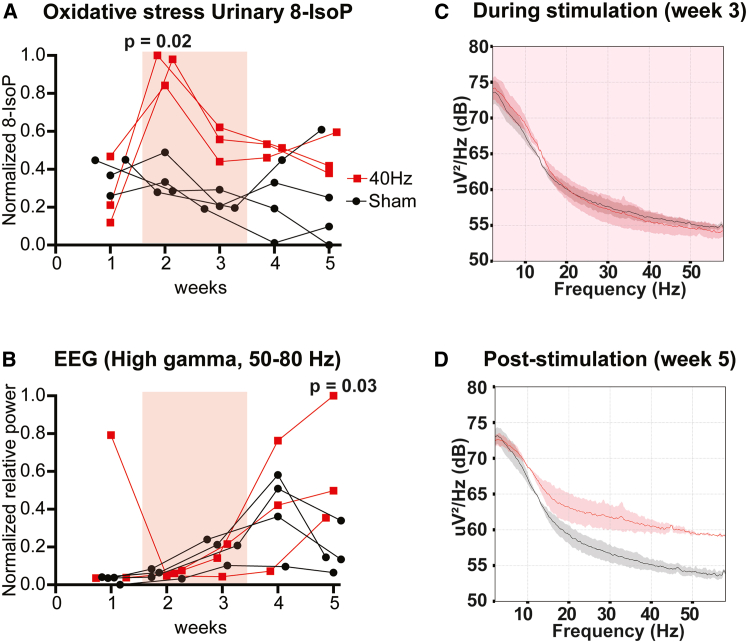
Redox imbalance precedes EEG changes in mice (A and B) Longitudinal measures of oxidative imbalance (urinary 8-IsoP) (A) and EEG gamma relative power (B) across 5 weeks in treated (red) and sham mice (black), starting with baseline measures (no EMF) for the first week, followed by 2 weeks of ELF-EMF (pink shading) and 2 weeks with EMF-off. EEG and 8-IsoP measures were carried out at the end of each week. Note the immediate rise of 8-IsoP at week 2 corresponding to ELF-EMF treatment onset, followed by a gradual rise in EEG gamma power by week 5 in ELF-EMF-treated mice, not seen in sham controls (*p* = 0.03, respectively; one-sided Mann-Whitney test). (C and D) Excerpts of EEG power spectra across frequencies in ELF-EMF-treated and sham mice during (C) and after exposure (D) periods. In (A) and (B), data are presented as individual mouse tracks over 5 weeks. In (C) and (D), data are presented as Mean ± 1 standard deviation.

**Figure 4:**
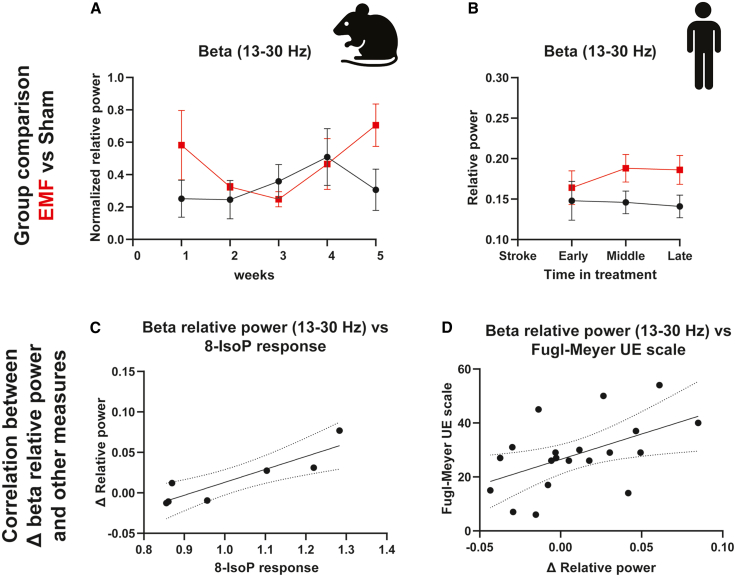
Elevated EEG beta power reflects earlier peripheral oxidative stress markers and concurrent stroke recovery (A) As observed for gamma power, EEG beta relative power gradually increases over several weeks of ELF-EMF treatment in mice. Data presented as Mean ± S.E.M. (B) Beta relative power at Cz was significantly higher in the ELF-EMF group than in the sham group in the middle and late bins (∗*p* = 0.05, one-tailed Mann-Whitney tests), while the decreasing trend in the sham group from middle to late was not statistically significant. (C) A significant correlation (R = 0.89, *p* = 0.007) between the peak 8-IsoP response at week 2 (relative to baseline) and EEG beta relative power change between weeks 2 and 5 in mice. Data are presented as individual mouse measurements, accompanied by a regression line and 95% confidence intervals. (D) Δ beta relative power (averaged over 3 sessions) correlated with Δ Fugl-Meyer UE scale (rs = 0.47, *p* = 0.03, *n* = 20). Data are depicted as individual patient measurements, accompanied by a regression line and 95% confidence intervals.

To explore the correlation of oxidative stress trajectories with electrical brain activity, we turned to electroencephalography (EEG). Mice were implanted with cranial adaptors for telemetric EEG transmitters and recorded weekly. Urine samples were collected in parallel for peripheral 8-IsoP measures of oxidative stress. In the same week ELF-EMF was initiated (week 2), we observed an increase in 8-IsoP levels (Figure 3A; samples collected on the last day of the week). We found a delayed increase in relative beta and gamma power 3 weeks post-ELF-EMF exposure (week 5; Figures 3B–3D), whereas urinary 8-IsoP decreased back to initial values at this time point (Figure 3A). The acute 8-IsoP level rise was correlated with the magnitude of increase in relative beta power four weeks after exposure began (R = 0.89, *p* = 0.007), indicating that 8-IsoP may potentially serve as an early biomarker for EEG response to ELF-EMF (Figure 4C).

### Stroke recovery reflects delayed neural activity changes

Finally, we assessed the functional relevance of ELF-EMF exposure to humans. We analyzed EEG from a double-blind, sham-controlled randomized clinical trial of an electromagnetic network targeting field (ENTF) therapy, an ELF-EMF treatment, in patients with post-stroke (Table 1). Similar to that which we have observed in mice, ENTF therapy produced a significant change in human EEG power four weeks after the start of treatment (Figure 4B).

**Table 1:** Participant demographics

	Sham Group (*n* = 8)	ENTF Group (*n* = 13)	All (*n* = 21)
Age, yrs, mean (±SD)	55.3 (±10.1)	54.3 (±17.8)	54.7 (±15.0)
Sex, female (%)	25%	15%	19%
Race-Ethnicity, South-Asian (%)	100%	100%	100%
Hand dominance, right (%)	100%	100%	100%
Affected hand, right (%)	63%	38%	48%
Time from stroke onset to first treatment, days, median (IQR)	14.0 (10.8–16.0)	9.0 (7.0–14.0)	11.0 (8.0–15.0)
FMA-UE Baseline, mean (±SD)	18.8 (±8.7)	26.8 (±11.5)	23.7 (±11.0)
mRS Baseline, mean (±SD)	3.4 (±0.7)	3.6 (±0.5)	3.5 (±0.6)

No significant differences were noted between groups at baseline.

Relative to the sham group, behavioral results in the treatment group showed greater improvement in the Fugl-Meyer Assessment - Upper Extremity (FMA-UE), with additional significant outcomes measured in several secondary endpoint measures, including the modified Rankin Scale (mRS), Box & Blocks Test (BBT), and the Action Research Arm Test (ARAT) pinch subscale.^9^ Notably, the FMA-UE score improvement was correlated with the degree of EEG beta power enhancement at the end of the trial (Figure 4D).

## Discussion

Our study elucidates a mechanism by which homogeneous ELF-EMF modulates brain function in a frequency-dependent manner, underscoring the need to tailor stimulus parameters. A poor understanding of the impact of ELF-EMF on brain biology has limited its application in clinical practice to date. In this study, we focused primarily on the gamma frequency range (30-80Hz), because recent findings indicate this oscillatory rhythm serves as a natural, reliable biomarker of an open plastic state characteristic of critical periods in brain development.^14^

We find that chronic exposure to 40 Hz ELF-EMF modulates oxidative stress in a time-dependent manner. Acute exposure triggers an initial rise in oxidative stress, as reflected by glutathione redox state (GSH/GSSG ratio) and urinary 8-IsoP levels. However, with the increased duration of exposure, these redox dynamics reverse, indicating a neuroprotective response. Unlike other methods of direct neurostimulation (tACS, TMS), changes in electrical brain activity (EEG) emerge only gradually in register with the chronic neuroprotective phase.

Accurate reflection of brain redox changes in the periphery can potentially serve as an early biomarker for EEG response to ELF-EMF. The magnitude of beta relative power increase was both predictive of functional stroke recovery in humans and predicted by the initial rise in redox imbalance in mice. Furthermore, frequency-specific ENTF therapy was shown to promote an improvement in upper extremity motor function; such functional improvement was positively correlated with a reversal of stroke-related changes in beta relative power.

These findings reveal cellular redox regulation to be a primary target of action for ELF-EMF that supports brain plasticity. This suggests a nuanced magneto-receptive cellular signaling process by which ELF-EMF impacts brain function. Low-intensity electromagnetic fields have been reported to induce human cryptochrome to modulate intracellular reactive oxygen species and aid neural circuit repair.^15^^,^^16^

Our results suggest that frequency-specific ELF-EMF induces a transient elevation in oxidative stress, which may trigger a homeostatic neuroprotective response with chronic exposure. This homeostatic response might play a role in synaptic changes.^17^^,^^18^ Therefore, precise investigation of the EEG sequelae associated with initial biomarkers of redox dysregulation may allow us to tailor optimal treatment protocols for neurotrauma and neurodegeneration, as well as enhance wellness.

### Limitations of the study

Our study provides promising evidence that ELF-EMF exposure holds frequency-specific neuroprotective benefits, including redox regulation in the mouse brain and stroke recovery in patients. However, direct tracking of oxidative stress modulation by ELF-EMF in human subjects (e.g., urinary 8-IsoP) with respect to later emergent EEG changes is still needed to potentially link these as biomarkers for recovery. Additional work remains to elucidate the underlying cellular mechanisms by which ELF-EMF exerts its sequential frequency-specific effects, which is crucial for developing optimally effective and precise treatment strategies for neurological conditions.

## Resource availability

### Lead contact

Those seeking additional information should contact the lead contact, who will fulfill their request, Takao K. Hensch (hensch@mcb.harvard.edu).

### Materials availability

Materials are available either from commercial resources (see above) or from our laboratory upon request.

### Data and code availability

• Raw data statements: All data reported in this article pertinent to animal studies will be shared by the lead contact upon request. For human data, due to patient identity and data collection under proprietary conditions, further discussion and investigator discretion will be needed upon request.
• All original code has been deposited at GitHub and will be shared upon request.
• Any additional information required to reanalyze the data reported in this article is available from the lead contact upon request.

## Acknowledgments

We thank Cameron Oram, Darred Surin, Alex Newman, and Kate Hoffman for mouse breeding and maintenance. We thank Natan Bornstein, Jeffrey L Saver, Arielle Hochberg, Dharam P Pandey, and Atul Prasad for their contributions to clinical trial design. Funded in part by BrainQ Technologies and WPI-IRCN (10.13039/501100001691Japan Society for the Promotion of Science).

## Author contributions

H.H.C.L., S.R.B., B.W., T.K.H., Y.S., and E.S. designed the study; H.H.C.L. and N.W.H. performed and analyzed mouse experiments; S.R.B. analyzed human and mouse data; H.H.C.L., S.R.B., T.K.H., A.P., and B.W. wrote the article; all authors reviewed and revised it.

## Declaration of interests

S.R.B., B.W., G.M.D., A.P., E.S., and Y.S. are employees of BrainQ Technologies, Ltd. H.H.C.L. is Co-Founder of Galibra Neuroscience, Inc., Galibra Neuroscience did not sponsor or participate in this study.

## STAR★Methods

### Key resources table

**Table:** 

REAGENT or RESOURCE	SOURCE	IDENTIFIER
**Critical commercial assays**		

8 Isoprostane ELISA kit	Abcam	Cat#Ab175819

**Experimental models: Organisms/strains**		

C57BL/6J mice	JAX	Cat#000664

**Software and algorithms**		

GraphPad Prism 9.3.1	GraphPad	RRID:SCR_002798
Python Programming Language (python 3)	https://www.python.org/	RRID:SCR_008394

### Experimental model and study participant details

#### Animals

All mice were housed in the animal facility of Boston Children’s Hospital in accordance with guidelines regulated by IACUC. Adult (>P60) male C57Bl/6J mice were used to minimize physiological variabilities due to potential asynchronized estrous stages in a female cohort. All experimental and control animals were age-matched for comparison and housed 3–5 per cage and provided with food and water *ad libitum*. Rodent diet is standardized, containing Prolab Isopro RMH 3000, gamma-irradiated to ensure bacteria-free condition before use.

#### Human subjects

The clinical trial was conducted at the BLK Super Specialty Hospital, New Delhi, India, a multi-specialty private hospital accredited by the Joint Commission International. Study operations were overseen by JSS Medical Research, an international, full-service contract research organization. The hospital institutional review board provided ethics approval, and written informed consent was obtained from all participants or their legal proxy. Additional information is available in the relevant manuscript (Weisinger et al., 2022).

### Method details

#### ELF-EMF exposure (mice)

The ELF-EMF device consisted of a pair of 56-turn Helmholtz coils (42 cm radius), capable of generating 1–100 Hz EMF fields at intensities of 0.3 to 10G produced by a BK Precision 4045B sine wave generator amplified by a dedicated amplifier. Mice (up to five per cage) behaved freely within their home cage placed on an acrylic platform centered between the coils, such that the entire cage was situated within a homogeneous EMF in the center of the coils without restriction to specific body or brain orientation. All metal mesh components inside the cages were removed to avoid interference of the homogeneous ELF-EMF permeating the cage and animals within the device. Duration of ELF-EMF exposure varied according to experimental conditions. Frequency of ELF-EMF was set to one of 7.6, 40 or 60Hz. Strength and stability of ELF-EMF were monitored throughout exposure sessions by ELF-EMF detector probe and Gauss meter. After ELF-EMF exposure, mice were returned to their typical housing environment. Control mice were placed at the same time 12 feet outside the experimental procedure room, where no ELF-EMF field from the device was detectable.

#### Oxidative stress measurement in mice

Mice were briefly anesthetized using 3% isoflurane quickly followed by decapitation. Cortex was freshly dissected and washed in phosphate-buffered saline (PBS; pH 5.5). A 10% homogenate was made in PBS (pH 5.5) and sonicated (15 s on ice). 100 μL of sonicated product was removed and used to determine protein content. 200 μL of the remaining product was added to 50 μL of 0.4N perchloric acid and spun at 13000 RPM on a tabletop microcentrifuge for 60min at 4°C to remove protein and cellular debris. 10 μL of sample was injected onto an Agilent Eclipse XDB-C8 (3 × 150 mm, 3.5 μm) reverse-phase C8 column using a Beckman System Gold HPLC consisting of a dual pump (model 125), autosampler (model 508) and an ESA CouloChemIII running a BDD analytical cell (model 5040) electrochemical detector at an operating potential of 1500 mV. A dual mobile phase gradient elution was used to resolve the analytes, consisting of mobile phase containing sodium phosphate (25 mM) and 1-octanesulfonic acid (2.1 mM), adjusted to pH 2.65 with phosphoric acid, with the second mobile phase (B) containing 50% acetonitrile. The system was run at 1 mL/min flow rate and ambient temperature with following gradients: 0 to 8min 0% B, 8 to 20min, gradient to 30% B. The system was allowed to equilibrate at 0% B from 25 to 36min. Peak area analysis was performed with 32-Karat software (Beckman Coulter; v8.0) based on standard curves generated for each compound. Samples normalized against protein content.

#### EEG acquisition and analysis (mouse)

All surgery and post-operation procedures were conducted in accordance with IACUC guidelines. Adult mice (>P60) were anesthetized by 1–2% isoflurane and head-fixed in a stereotaxic frame. Hair was removed from between the ears forward, and an incision made along the midline to expose the cranial bone underneath. Three burr holes were made with a bone drill corresponding to the somatosensory region bilaterally (bregma −2.5 mm, midline ±1.5 mm) and right visual cortex (bregma -4mm, midline +1.5 mm). A three-pronged electrode mount (Plastics1) was then implanted with electrode leads lowered to 0.5 mm below the pial surface, then secured by dental cement. Once solidified, the skin was sutured to cover the head mount held by further application of VetBond. Mice were singly housed and returned to their home cage after surgery. One week later, implanted mice were subjected to telemetric EEG recording using the BIOPAC epitel system. Mice were briefly anesthetized by 1% isoflurane (<30s) to allow time to connect a wireless EEG transmitter to the electrode mount implant. Mice were then placed inside a plastic, rectangular recording chamber with bedding, food and water. A camera was used to further monitor mouse activity and continuous telemetric EEG recording was initiated 10min after habituation to the recording arena.

The total study duration was 5 weeks: EEG was collected 3 times a week and IsoP once a week (immediately after EEG session) throughout the experiment controlling for experimental procedures and data collection cadence. Experiments were conducted in a temperature-controlled environment to avoid potential physiological impacts due to temperature differences across the experimental paradigm. EEG recordings and IsoP sampling from week 1 were used to establish the baseline, followed by 2 weeks of ELF-EMF stimulation. The study continued for an additional 2 weeks of follow-up after ELF-EMF stimulation (weeks 4 and 5).

Analysis of continuous EEG data was performed using the open-source MNE package. Signals were band-pass filtered at 0.5–80 Hz. Power line components were attenuated by a notch filter at 60 Hz. The first 10 min and last 10 s of each recording were excluded from the analysis to avoid artifacts. The filtered signal was segmented into 5-s epochs and baseline correction was applied by subtraction of the average of the preceding epoch.

The spectral analysis included the calculation of the Power Spectral Density (PSD) using the multi-taper method and extraction of relative power of the bands of interest (beta, 13–30 Hz; lower gamma, 30–50 Hz; higher gamma, 50–80 Hz). Bad epochs were identified using the COPOD algorithm (applied over feature space) and removed. The number of artifact-free epochs retained per mouse for each week was 360 ± 60 (80% of all epochs). An average of each feature was calculated for each electrode and week. Averaged features were then compared between groups using the Mann-Whitney test.

#### Urinary 8-isoprostane (8-IsoP) detection

Mice were placed in a circular polypropylene arena of 8-inch diameter allowing free exploratory movement with clean parafilm mounted on the floor. After voluntary urination (100 μL in volume, up to 1.5 h in the arena to avoid prolonged stress), mice were returned to their home cage. Urine samples were collected from the parafilm into 1.5 mL sample collection tubes for subsequent 8-IsoP detection using a commercial ELISA kit (Abcam).

#### ELF-EMF exposure (human)

We analyzed EEG from a double-blind, sham-controlled randomized clinical trial of ELF-EMF treatment in patients in the subacute phase after ischemic stroke.^9^ Participants were randomized to receive Electromagnetic Network Targeting Field therapy (ENTF) (*n* = 13) or sham (*n* = 8) treatment in conjunction with 10 min of physical therapy; treatment was administered with a proprietary stimulation device (BQ 1.0; BrainQ Technologies Ltd., Jerusalem, Israel), exposing the entire brain and the cervical and upper thoracic portion of the spinal cord to the ENTF. The device technology uses machine learning algorithms to identify high-resolution spectral patterns that characterize motor functions within EEG measurements recorded during functional motor tasks, as previously described.^9^

Exposure to ELF-EMF therapy sessions lasted for 40 min, 5 days/week over 8 weeks (40 sessions in total). Continuous EEG was recorded from all participants approximately twice a week for 8 weeks. EEG recording did not take place during ENTF exposure. During EEG recording, participants were asked to perform alternating grip, reach and rest blocks (for more details, please see Weisinger et al., 2022). Functional endpoint measures included the Fugl-Meyer Assessment - Upper Extremity (FMA-UE; primary), as well as a wide battery of secondary endpoint measures including the modified Rankin Scale (mRS), the Action Research Arm Test (ARAT), the Box & Blocks Test (BBT), the Fugl-Meyer Assessment – Lower Extremity (FMA-LE), the modified Rankin Scale (mRS) of global disability, the National Institutes of Health Stroke Scale (NIHSS), the Patient-Reported Outcome Measurement Information System Global 10 (PROMIS-10), Notably, some prespecified outcome measures were not analyzed due to <80% valid data. These include: cognitive measures [Trail Making Test; Montreal Cognitive Assessment], as they were administered in English, which was not most participants' primary language; imaging (MRI), because of variability in scan parameters due to use of multiple scanners; and blood biomarkers, as some growth factors were out of the detection range for many subjects.

#### EEG acquisition and analysis (human)

EEG was recorded using a dry electrode cap (ANT Neuro, Hengelo, The Netherlands). The cap contained 8 data channels (F3, Fz, F4, C3, Cz, C4, P3, P4) with ground (A1) and reference ear-clip (A2) electrodes. Due to poor signal quality of the frontal electrodes, only data from central and parietal electrodes were analyzed.

Participants sat comfortably facing a wall, focusing on a marked fixation point throughout the session. A session comprised four different parts each lasting 2 min and 40 s. Each session included the following four motor tasks: right-hand grip, right-hand reach, left-hand grip, and left-hand reach. Each task consisted of eight 20-s blocks that alternated between motion and rest. Each block began with a brief beep. During the motion blocks, participants were instructed to perform a repetitive grip or reach motion, respectively, at a steady pace (∼3 s per movement). During the rest blocks, participants were instructed to relax (while keeping eyes open) and remain as still as possible.

EEG from the (20-s) rest blocks was analyzed after removing the first and last 2 s, due to artifact contamination during these segments. EEG was then downsampled to 256 Hz, and a zero-phase finite impulse response bandpass filter (0.5–30Hz) was applied, as implemented in mne-python.^19^ On this data, we ran an artifact subspace reconstruction (ASR) procedure^20^ to eliminate high amplitude noise. Data for channels identified as flat or, alternatively, contaminated by high amplitude noise was interpolated (spherical spline interpolation). Independent component analysis (ICA) was used to extract components of which some were rejected after employing the multiple artifact rejection algorithm (MARA).^21^

Relative band power features were calculated for four spectral bands (delta 0.5-4Hz, theta 4-8Hz, alpha 8-13Hz, beta 13-30Hz). Features were averaged across blocks, yielding an average of each feature for each session. Outlier removal for the averaged features was by the COPOD algorithm,^22^ with the constraint that data from 80% of the sessions be retained. Participants with missing data for more than half of the sessions were excluded from analysis.

### Quantification and statistical analyses

#### Statistical analysis

Individual parameters, two-group *t* test were performed using Graph-pad Prism version 9.3.1 for Windows, GraphPad Software, San Diego, California USA, www.graphpad.com. Mann-Whitney tests were performed for group comparisons of EEG features and urinary 8-IsoP levels. Spearman’s rho correlation analysis was performed to examine the correlation between EEG measures and Fugl-Meyer upper extremity (UE) scores. As the Fugl-Meyer scale is ordinal in nature, Spearman’s rank correlation coefficient is an appropriate choice for analyzing the correlation with EEG relative power. Additionally, Pearson correlation analysis was employed to assess the relationship between EEG features and IsoP levels. EEG processing and statistical analysis were performed using python 3 and Graph-pad Prism 9.3.1.

### Additional resources

#### Clinical trial

Part of the data used in this manuscript originated from the clinical trial NCT04039178, where efficacy of a non-invasive device with low-intensity electromagnetic field dedicated treatment to enhance the recovery of stroke patients' impaired hand motor function was tested.

Description: https://clinicaltrials.gov/study/NCT04039178?term=brainq&rank=1.
